# Biomaterials for immunomodulation in wound healing

**DOI:** 10.1093/rb/rbae032

**Published:** 2024-03-27

**Authors:** Ying Wang, Katrina Vizely, Chen Yu Li, Karen Shen, Amid Shakeri, Ramak Khosravi, James Ryan Smith, Eugene Alfonzo I I Alteza, Yimu Zhao, Milica Radisic

**Affiliations:** Institute of Biomedical Engineering, University of Toronto, Toronto, ON M5S 3G9, Canada; Toronto General Research Institute, University Health Network, Toronto, ON M5G 2C4 Canada; Department of Chemical Engineering and Applied Chemistry, University of Toronto, Toronto, ON M5S 3E5, Canada; Department of Chemical Engineering and Applied Chemistry, University of Toronto, Toronto, ON M5S 3E5, Canada; Toronto General Research Institute, University Health Network, Toronto, ON M5G 2C4 Canada; Institute of Biomedical Engineering, University of Toronto, Toronto, ON M5S 3G9, Canada; Toronto General Research Institute, University Health Network, Toronto, ON M5G 2C4 Canada; Toronto General Research Institute, University Health Network, Toronto, ON M5G 2C4 Canada; Division of Cardiovascular and Thoracic Surgery, Department of Surgery, Duke University Medical Center, Durham, NC 27710, USA; Institute of Biomedical Engineering, University of Toronto, Toronto, ON M5S 3G9, Canada; Institute of Biomedical Engineering, University of Toronto, Toronto, ON M5S 3G9, Canada; Institute of Biomedical Engineering, University of Toronto, Toronto, ON M5S 3G9, Canada; Toronto General Research Institute, University Health Network, Toronto, ON M5G 2C4 Canada; Institute of Biomedical Engineering, University of Toronto, Toronto, ON M5S 3G9, Canada; Toronto General Research Institute, University Health Network, Toronto, ON M5G 2C4 Canada; Department of Chemical Engineering and Applied Chemistry, University of Toronto, Toronto, ON M5S 3E5, Canada; Terrence Donnelly Centre for Cellular & Biomolecular Research, University of Toronto, Toronto, ON M5S 3E1, Canada

**Keywords:** immunomodulation, peptide-based biomaterials, innate immunity, angiogenesis for wound healing, cellular response

## Abstract

The substantial economic impact of non-healing wounds, scarring, and burns stemming from skin injuries is evident, resulting in a financial burden on both patients and the healthcare system. This review paper provides an overview of the skin’s vital role in guarding against various environmental challenges as the body’s largest protective organ and associated developments in biomaterials for wound healing. We first introduce the composition of skin tissue and the intricate processes of wound healing, with special attention to the crucial role of immunomodulation in both acute and chronic wounds. This highlights how the imbalance in the immune response, particularly in chronic wounds associated with underlying health conditions such as diabetes and immunosuppression, hinders normal healing stages. Then, this review distinguishes between traditional wound-healing strategies that create an optimal microenvironment and recent peptide-based biomaterials that modulate cellular processes and immune responses to facilitate wound closure. Additionally, we highlight the importance of considering the stages of wounds in the healing process. By integrating advanced materials engineering with an in-depth understanding of wound biology, this approach holds promise for reshaping the field of wound management and ultimately offering improved outcomes for patients with acute and chronic wounds.

## Graphical abstract

**Figure:**
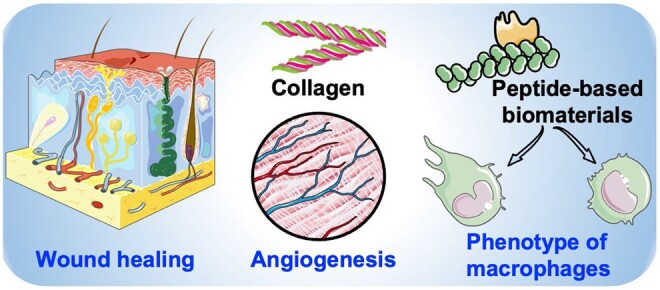

## Introduction

The skin, recognized as the body’s largest organ, plays a pivotal role as a robust barrier, defending against a myriad of threats. These include safeguarding against microbial invasion, providing resilience against mechanical damage, regulating exposure to extreme temperatures, and shielding the body from potentially harmful ultraviolet radiation. Thus, the skin establishes and preserves the optimal microenvironment essential for the effective functioning of internal organs. The significance of skin becomes evident when considering the substantial costs associated with injuries and the healing process, which include both direct medical expenses and indirect costs such as lost productivity and long-term care. In the USA, non-healing wounds, scarring, and burns present significant challenges for both patients and the medical system, resulting in annual expenditures of $50 billion, $12 billion, and $7.5 billion, respectively [1]. A comprehensive understanding of wound-healing mechanisms and the active promotion of efficient treatment can play a pivotal role in reducing these expenses and ultimately enhancing overall healthcare outcomes.

Acute wounds generally follow a predictable and orderly healing process consisting of well-defined stages including hemostasis, inflammation, proliferation, and remodeling, while chronic wounds fail to progress through the normal healing stages within the expected timeframe. Immunomodulation is crucial both in acute and chronic wound-healing processes. In the acute wound-healing process, the inflammatory phase begins following hemostasis. Immune cells, particularly neutrophils and macrophages, infiltrate the injured area to clear debris and bacteria and release various signaling molecules. The balanced and well-coordinated immune response at this stage helps initiate subsequent fibroblast proliferation and tissue remodeling. On the other hand, the dysregulated balance between pro-inflammatory and anti-inflammatory signals in chronic wounds that are associated with underlying health issues, such as diabetes, peripheral vascular disease and immunosuppression, hinders the normal healing stages. For example, the disordered metabolic pathways and impaired immune response in diabetic patients result in a delayed healing process at each stage. In addition, the hyperglycemia of diabetes creates a favorable environment for bacterial growth, resulting in increased susceptibility to infections as well as serious complications. While immunosuppression hinders the initiation of wound healing, excessive inflammation can lead to prolonged pain and swelling. As such, appropriate immunomodulation is a vital aspect of both acute and chronic wound healing. Therapies aimed at modulating the immune response can help promote a shift toward a more pro-healing environment.

Traditional wound-healing approaches have evolved into two primary strategies: one involves establishing an optimal microenvironment for the wound-healing process, while the other centers on modulating cellular processes to facilitate effective wound closure. Advanced wound dressings, embedded with growth factors, cytokines, and immunomodulatory agents, have proven to improve the wound microenvironment by facilitating cell migration and reducing the risk of bacterial colonization. However, delayed wound closure and excessive scar tissue formation, known as hypertrophic scarring, may occur due to the dysregulated cellular processes. These can cause fibroblasts to produce excessive collagen, leading to raised and thickened scars that can limit mobility and cause discomfort. Efforts to modulate cellular activities by introducing growth factors, such as vascular endothelial growth factor (VEGF) and platelet-derived growth factor (PDGF), have shown the potential to accelerate wound closure. Nevertheless, the utilization of growth factors can cause significant costs and has been associated with an increased susceptibility to cancer development. For instance, the topical administration of REGRANEX gel containing becaplermin, a recombinant human PDGF-BB, initially demonstrated an increased rate of mortality secondary to malignancy in a retrospective cohort study for the treatment of lower extremity diabetic neuropathic ulcers; additional follow-up, however, indicated no elevated overall cancer mortality risk [2, 3]. Studies involving the administration of specific growth factors such as VEGF have exhibited remarkable effects in promoting angiogenesis and tissue regeneration, resulting in faster wound healing [4]. However, the uncontrolled stimulation of cellular proliferation by these growth factors has also raised concerns about their potential contribution to the formation of hemangioma-like lesions, emphasizing the intricate balance that must be struck when harnessing their therapeutic potential [5, 6].

Addressing this medical challenge requires the development of biomaterials to create an ideal macroscopic environment. These materials should not only optimize the overall healing conditions but also safely modulate the wound-healing process, aiming at achieving effective wound closure and minimizing scar formation [7]. Given the pivotal role of immunomodulation in mitigating persistent inflammation in both acute and chronic wounds, biomaterials can be leveraged for immunomodulation and the reduction of bacterial burden in the wound. Reactive oxygen species (ROS) produced from oxidative stress, increased expression of pro-inflammatory cytokines, and bacterial infection can cause the wound to become a continuous inflammatory microenvironment. Among numerous strategies, researchers can precisely tailor the immune response by designing biomaterials that control the release of anti-inflammatory compounds, possess inherent antimicrobial properties, promote nanomaterial penetration and present bioresorbable properties. Green biomaterials, synthesized or modified using alternative green and sustainable technologies like CO_2_ supercritical extraction, offer promising solutions for wound care by aiming to improve patient outcomes while reducing environmental impact [8]. Clinically, pearl powder promotes granulation tissue maturation, accelerates healing and minimizes scarring [9]. Compared to traditional water, acid or enzyme extraction methods, the CO_2_ supercritical extraction system provides faster extraction of active ingredients from pearl powder due to its higher permeation properties. Moreover, this method is environmentally friendly as CO_2_ is a nonhazardous solvent [10]. Thus, this approach is in line with the Green Biomaterials principle of the use of safe and greener solvents. By combining advanced biomaterials engineering with an in-depth understanding of wound biology, these strategies have the potential to revolutionize wound care and improve outcomes for patients with both acute and chronic wounds [6, 11, 12].

**Figure 1.**
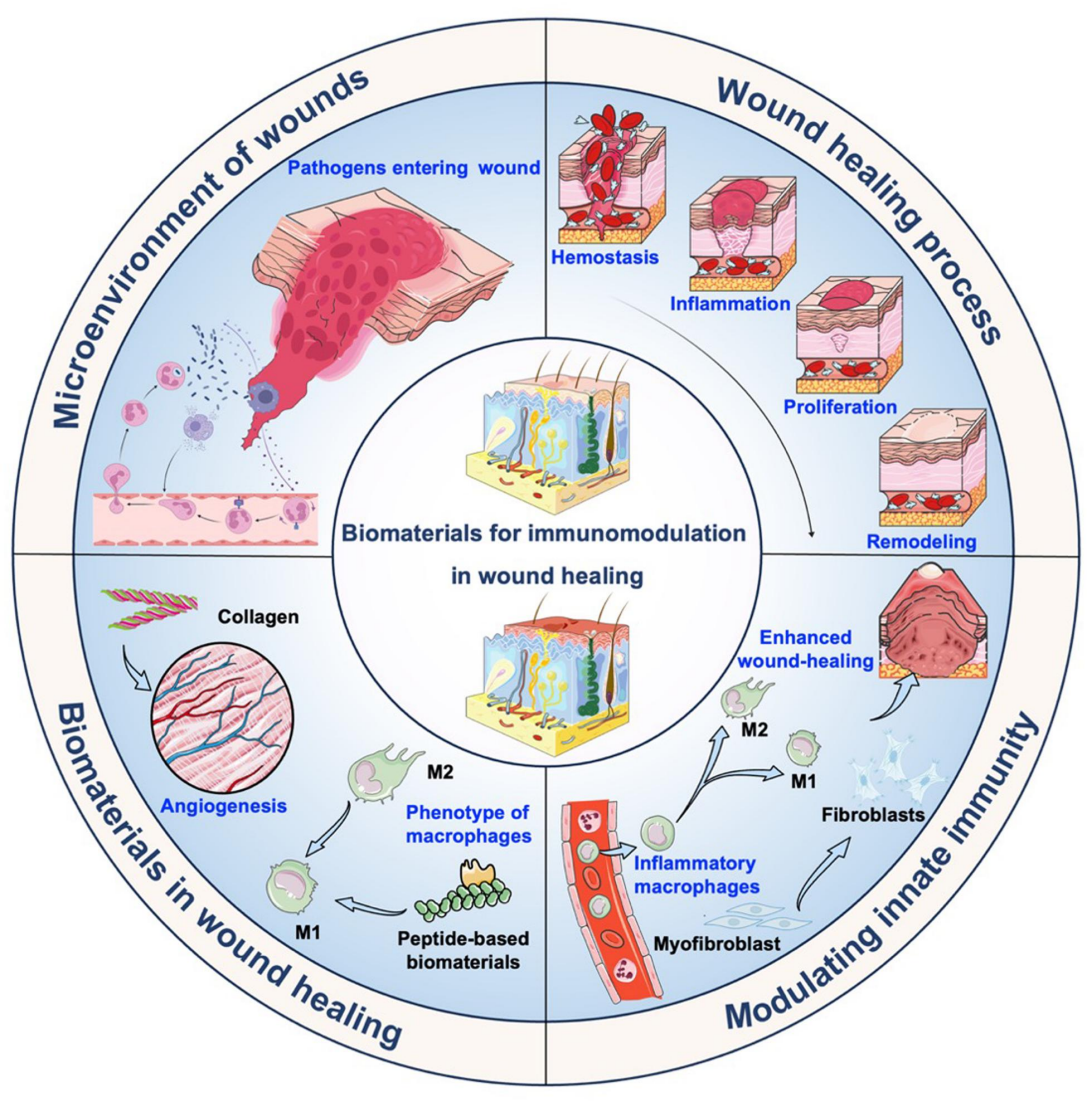
Schematic illustration of the biomaterials for immunomodulation in wound healing. The wound-healing process typically encompasses the sequential stages of hemostasis, inflammation, proliferation and remodeling. The innate immune system is the first line of defense and includes physical barriers like the skin, as well as cellular and molecular components like white blood cells, antimicrobial proteins and various signaling pathways. Emerging biomaterials present excellent opportunities for leveraging immunomodulation and reducing bacterial burden in wounds. Created with BioRender.com and Servier Medical Art.

In this review, we expand upon this interaction between the skin and recently developed biomaterials in the context of the wound-healing process (Figure 1). First, we present the intricate processes of wound healing, with an emphasis on the microenvironment of wounds and the significance of immunomodulation in both acute and chronic wounds. The dysregulated immune response in chronic wounds is delineated, as are its links to comorbidities like diabetes and immunosuppression. Additionally, traditional wound-healing approaches and recent peptide-based biomaterials are introduced to highlight the significance of an optimal microenvironment and cellular processes in facilitating wound closure. The review concludes with a forward-looking perspective on the potential of biomaterial development in revolutionizing wound care, particularly in the realm of immunomodulation, with a focus on regulating neutrophil and macrophage activities. The integration of advanced materials engineering and a profound understanding of wound biology holds promise for reshaping the field of wound management, offering improved outcomes for patients with acute and chronic wounds.

## Composition of healthy skin

The skin acts as our primary line of defense against the external environment, and while its characteristics vary across species, the fundamental structure remains largely consistent [13]. The outermost layer, the epidermis, is comprised of a stratified squamous epithelium of keratinocytes separated by a basal membrane. It also includes Langerhans cells (LCs) and melanocytes [14]. Providing structural elasticity, integrity and nutrition to the skin, the dermis constitutes the internal layer [15]. Additional structural elements in the dermis encompass sebaceous, apocrine and eccrine sweat glands, blood and lymphatic vessels, nerve endings as well as hair follicles extending from the epidermis [16].

### Epidermis

Being the outermost layer, the epidermis is frequently subjected to damage, leading to a continuous shedding of the uppermost cornified cells and a high turnover rate. This necessitates tightly regulated processes to uphold homeostasis. The homeostasis of the cells in the epidermis, such as keratinocytes, melanocytes, LCs and Merkel cells [14], enables a coordinated injury response. These cells express various pathogen recognition receptors (PRRs), which upon ligand recognition result in the initiation of downstream signaling pathways and the production of inflammatory cytokines, chemokines and antimicrobial molecules. Keratinocytes migrate, proliferate and differentiate to complete the re-epithelialization process and restore the epidermal barrier during skin repair and tissue maintenance [17]. Along with dermal fibroblasts, keratinocytes also participate in wound contraction [18]. LCs constitute a vital dendritic cell population crucial for preserving immune homeostasis in the skin through effective antigen presentation [19]. Specifically, keratinocytes produce inflammatory cytokines following tissue injury, such as interleukin (IL)-1β, tissue necrosis factor (TNF)-α, and Granulocyte-macrophage colony-stimulating factor (GM-CSF), leading to the migration of LCs to the lymph nodes. LCs play a pivotal role in priming naive T cells, acting as a bridge between the innate and adaptive immune systems in response to a wound [20]. Subsequently, activated T cells are recruited to the site of injury, leading to the emergence of two distinct populations of LCs: shortly thereafter, permanent LCs and newly formed monocyte-derived LCs [21].

Besides that, the resident stem cell populations within the epidermis play a crucial role in responding to damage and are essential for the regeneration and maintenance of the skin [22]. Typically, various structures within the epidermis, including the sebaceous glands, interfollicular epidermis, sweat glands and hair follicles, harbor their own resident stem cells. For example, tissue-resident multipotent stem cells (MSC) can promptly differentiate into epidermal cells and aid the wound-healing process. Additionally, stem cells derived from adipose tissue and bone marrow are popular choices for stem cell-based therapy due to their ability to readily differentiate into keratinocytes, endothelial cells, pericytes and monocytes [23]. The shift between these cellular roles is regulated by signaling molecules including cytokines, growth factors, matrix metalloproteinases (MMPs) and chemokines [24].

### Dermis

Dermal fibroblasts play a vital role in normal wound healing by supporting essential processes, including the degradation of fibrin clots, the formation of collagen structures and the deposition of new extracellular matrix (ECM) [25]. Fibroblasts are heterogeneous and dynamic, allowing them to contribute appropriately to the healing process [26]. However, variable cell surface markers impede the consistent identification and characterization of dermal fibroblasts [27]. There is evidence suggesting fibroblast subpopulations in the dermis exhibit a stratified organization, with distinct roles and locations within the tissue. In the upper dermis, fibroblasts are recruited during re-epithelialization, which is the process of regenerating the epithelial layer and is essential in the formation of hair follicles by creating a supportive microenvironment [28]. Lower subpopulations of fibroblasts are primarily involved in dermal repair following injury. These fibroblasts specialize in synthesizing ECM and interacting with adipocytes, demonstrating their significant role in initiating the injury response [29].

The heterogeneity of dermal fibroblast populations extends beyond their spatial distribution [30]. Fibroblasts and their lineage display remarkable plasticity, indicating their capacity to undergo diverse differentiation and maturation processes. For example, fibroblast maturation into myofibroblasts during wound-healing results in the production of regulatory molecules and crosstalk with other cell populations [31]. These discoveries offer novel insights into the dynamic nature of fibroblast subpopulations and their roles in maintaining tissue homeostasis and facilitating wound-healing processes.

### Immune cells

A pivotal aspect of the wound-healing process revolves around the recruitment of immune cells at the site of injury. These cells can either be resident in the tissue at the time of injury, such as resident macrophages, or recruited from the surrounding tissue to infiltrate the wound area. The proper coordination of immune cell activity is essential for an effective wound-healing response. For example, T-helper cells, a type of immune cell, signal epithelium regrowth and injury site resealing by producing the cytokine IL-22 [32]. Since immune cells are involved in various stages of the wound-healing process, a dysregulated immune response can evoke destructive consequences [33, 34].

In the early stages of wound healing, neutrophils are the first to be recruited from the bloodstream into the wound environment. Upon encountering infectious agents in the wound, neutrophils aggregate at the site to eradicate these pathogens through the generation of ROS, cytotoxic granules, neutrophil extracellular traps (NETs) and phagocytosis. It was reported that genetically modified cells with a reduction in neutrophil recruitment exhibit delayed wound healing [35]. However, this robust response aimed at combating microbial invaders can potentially result in excessive inflammation and impaired wound healing. Apoptotic neutrophils release signaling molecules such as phosphatidylserine that promote phagocytosis and subsequent interactions with macrophages, leading to the development of a repair macrophage phenotype characterized by the production of transforming growth factor-beta (TGF-β) and IL-10 [36]. For example, Jin’s group developed engineered neutrophil apoptotic bodies to mimic the role of apoptotic neutrophils in reprogramming macrophages and resolving inflammation [37]. Taken together, despite excessive neutrophil activity potentially impeding the healing process, their timely recruitment and subsequent interactions with other immune cells, such as macrophages, play essential roles in orchestrating tissue repair [38].

Macrophages play a dynamically coordinated role in the wound-healing process, acting as key contributors to the signaling cascade by producing and releasing various factors [39]. Previous studies have explored the function of macrophages in various stages of wound healing over time [40], utilizing gene expression to characterize their functions [41]. In the early stages of healing, macrophages exhibit a pro-inflammatory phenotype, marked by the release of inflammatory cytokines, such as TNF-α, IL-1 and IL-6, ROS, and nitric oxide [42, 43]. As the wound environment evolves, macrophages shift their phenotype to support angiogenesis and ECM deposition [44, 45]. Key mediators in this transition include VEGF-A, insulin-like growth factor 1, PDGF and TGF-β, along with the MMP inhibitor tissue inhibitor of metalloproteinases 1 [45–47]. In the later stages of healing, there is a suppression of the inflammatory response with the release of IL-10 [48].

Besides neutrophils and macrophages, dendritic cells are key players in the immune system, serving as crucial links between innate and adaptive immune responses [49]. Dendritic cells are equipped with various pattern-recognition receptors that allow them to sense and respond to signals associated with tissue damage or infection. Upon detecting injury to the skin, dendritic cells capture antigens from the damaged tissue, including debris and molecules associated with the injury [50]. Activated dendritic cells migrate to nearby lymph nodes, where they present the processed antigens to T cells. This presentation occurs through the interaction of antigen-MHC complexes on the dendritic cell surface with T-cell receptors [50]. Activated T cells, in turn, release cytokines, such as interferon-gamma (IFN-γ) [51], programmed death-1 (PD-1) [52], and TNF-β [53, 54], that further regulate the immune response. These mutual interactions between dendritic cells and T cells are crucial for initiating an adaptive immune response. Beyond their role as antigen-presenting cells, dendritic cells play a role in regulating the balance between pro-inflammatory and anti-inflammatory signals by releasing cytokines and interacting with other immune cells involved in the wound-healing process. For example, dendritic cells can stimulate the activation and recruitment of fibroblasts, which are responsible for collagen deposition and tissue reconstruction [55]. In summary, dendritic cells act as dynamic regulators of the inflammatory response during wound healing. Through the release of cytokines, recruitment of immune cells, and interaction with T cells, dendritic cells contribute to the maintenance of a balanced and controlled inflammatory environment, ensuring effective tissue repair and recovery.

## The stages and microenvironment of the healing wound

### Stages of wound healing

**Figure 2.**
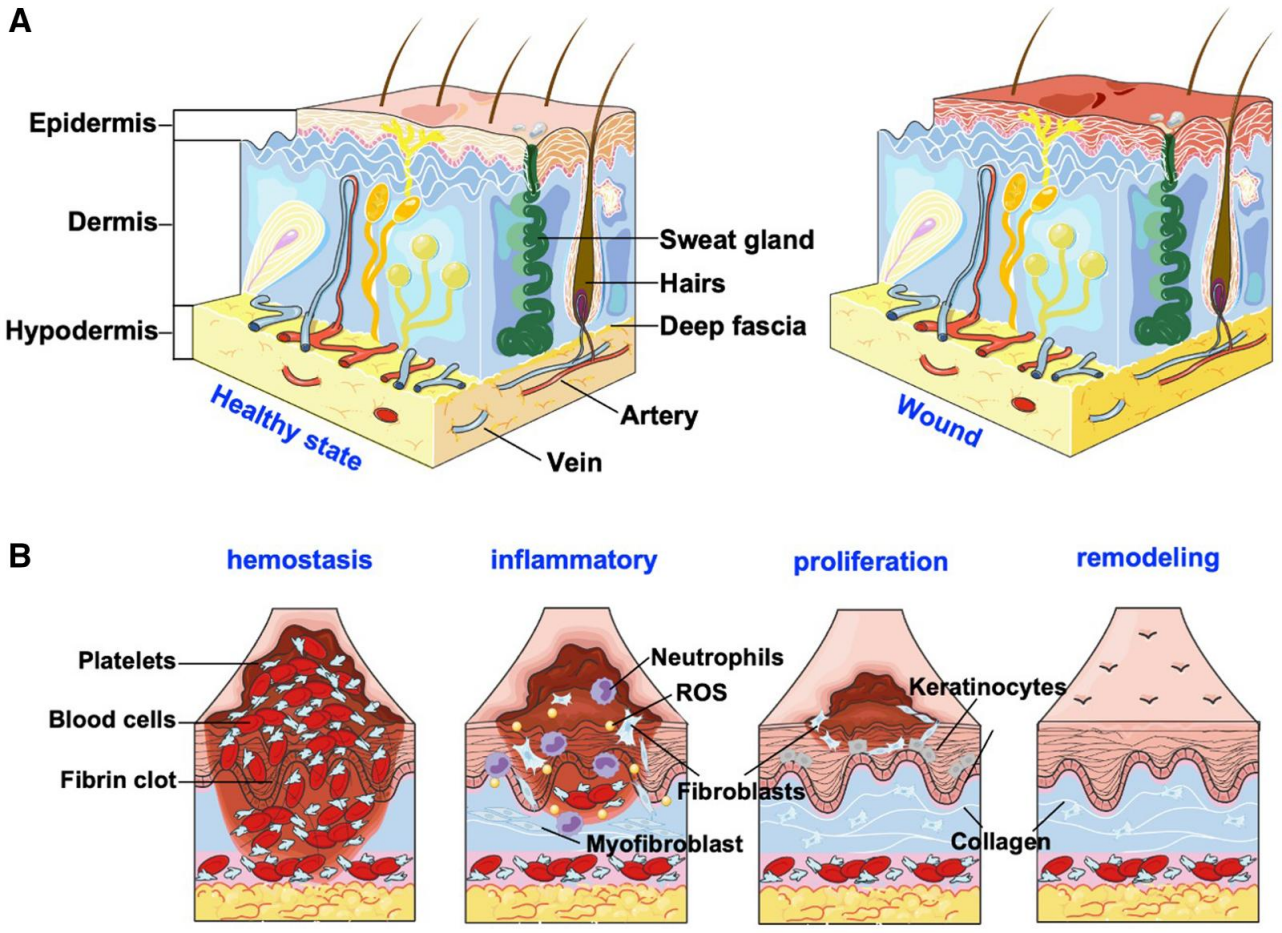
The microenvironment of the skin in both its healthy and wounded states, along with the various stages of the wound-healing process. (**A**) Schematic representation of the skin in healthy and wound states, respectively. (**B**) The process of wound healing, including hemostasis, inflammation, proliferation and remodeling. Created with Servier Medical Art templates. Created with Servier Medical Art.

A wound is generated when the normal tissue anatomy is disrupted, which is categorized as acute or chronic, depending on the type of repair process involved [15]. Acute wounds are distinguished by complete healing with minimal or no scar formation (Figure 2A). Following injury, four major overlapping phases necessitate precise cellular coordination for effective healing: hemostasis, inflammation, proliferation, and remodeling (Figure 2B) [6].

In the initial stage of wound healing, hemostasis, the cessation of bleeding is imperative after vascular damage. Initially, vasoconstriction restricts blood entry to the site. Subsequently, primary and secondary hemostasis mechanisms involve the recruitment of platelets and clotting factors, including fibrinogen. These processes run concurrently, culminating in the formation of a platelet plug and fibrin mesh, effectively arresting the bleeding. This event releases complement and growth factors, establishing provisional scaffolding essential for the infiltration of cells necessary for wound healing [56].

The inflammatory phase occurs within the first 72 h, overlaps with hemostasis, and is characterized by cellular signals that recruit pro-inflammatory immune cells. In response to injury, host cells release lipid mediators, chemokines, damage-associated molecular patterns (DAMPs), and hydrogen peroxide to recruit neutrophils and other inflammatory cells, facilitating the elimination of pathogens [42, 57]. This is followed by the activation of local macrophages in the skin by danger signals and the recruitment of monocytes from the circulation. In the wound, DAMPs, pathogen-associated molecular patterns, and interferon-γ (IFN-γ) initiate the differentiation of monocytes into a pro-inflammatory phenotype (M1) [58, 59]. M1 macrophages eliminate pathogens and cellular debris via phagocytosis and release pro-inflammatory cytokines and chemokines to activate natural killer cells, macrophages, and helper T cells [39]. Pro-inflammatory cytokines also drive the migration of endothelial cells, epithelial cells, and fibroblasts to the wound site [60].

The third phase of wound healing, known as the proliferative phase, unfolds within 3–10 days following injury. Cytokines, growth factors, interleukins, and angiogenic factors are involved in the formation of granulation tissue, which fills up the wound area [58]. A temporary ECM is established by the influx of endothelial cells, keratinocytes, and fibroblasts depositing collagen. Angiogenesis, the formation of new blood vessels from endothelial cells, is facilitated by growth factors produced by M2 macrophages [38, 39]. Re-epithelization involves cytokines and growth factors stimulating basal keratinocytes to accumulate to close the wound surface.

The remodeling phase can last months or even years following injury. In granulation tissue, fibroblasts differentiate into myofibroblasts, which drive wound contraction through the expression of smooth muscle actin. This phase is marked by the degradation of the ECM through the action of MMPs and TIMPs. This process paves the way for the regression of neovessels and the deposition of more resilient type I collagen fibers. Type I collagen replaces type III collagen, the main component of granulation tissue. Lastly, as blood flow and angiogenesis slow down, myofibroblasts and vascular cells undergo apoptosis [58].

### Cytokine microenvironment

The maintenance of barrier and mechanical qualities in the epidermis and dermis heavily relies on the contributions of keratinocytes and fibroblasts [61]. Keratinocytes and fibroblasts interact cyclically and intricately, resulting in effective wound closure and tissue remodeling [62, 63]. Through signaling cytokines, fibroblasts and keratinocytes play critical roles in the immunological response by carrying specialized PRRs on their surfaces, such as RIG-I-like receptors, NOD-like receptors and Toll-like receptors [64]. These PRRs aid in the recognition of patterns in pathogens such as bacteria, viruses and fungi, as well as endogenous chemicals generated during tissue injury or inflammation. When PRRs attach to injured tissue, they initiate intracellular signaling cascades, one of which is the nuclear factor kappa-light-chain-enhancer of activated B cells (NF-κB) pathway [65]. In resting cells, an inhibitory protein termed IB (inhibitor of B) stores NF-κB in the cytoplasm. Signals from PRRs cause IB to degrade, allowing NF-B to translocate into the nucleus and bind to certain DNA sequences (B sites). This increases the transcription of numerous genes, including those responsible for cytokine production. These cytokines are then packed into vesicles before being transported to the cell membrane and secreted into the extracellular environment by exocytosis. Cytokines then interact with nearby immune cells, such as lymphocytes, macrophages and dendritic cells, by binding to specific receptors on their surface. The activated intracellular signaling in immune cells would result in a variety of responses, such as immune cell activation, proliferation, differentiation and migration [57]. Together, the cytokines released by fibroblasts and keratinocytes have an impact on the nature of the immune response, influencing aspects such as inflammation, tissue healing and immune cell recruitment.

TGF-β is a major cytokine that is produced by keratinocytes and fibroblasts. The main purpose of TGF-β is to encourage fibroblasts to produce ECM components such as collagen, fibronectin and proteoglycans, which are essential for tissue integrity and granulation tissue development during wound healing [66]. TGF-β also stimulates fibroblasts, causing them to differentiate into myofibroblasts [67]. Myofibroblasts have contractile capabilities and are important for wound contraction, which reduces wound size throughout the healing process; however, overactivation can lead to scarring. Furthermore, this cytokine promotes angiogenesis by encouraging the migration and proliferation of endothelial cells, promoting the formation of new blood vessels in the wound bed to ensure an adequate blood supply for delivering oxygen and nutrients to the healing tissue [68].

IL-1 is another important cytokine involved in wound healing. By generating an inflammatory response and coordinating multiple cellular processes, IL-1 plays an important role in tissue repair and wound healing. When tissue is injured, resident cells release IL-1, which acts as an alarm signal that recruits immune cells, particularly neutrophils, to the wound site [59]. Neutrophils are crucial in the early phases of wound healing because they eliminate debris and pathogens, generating an environment favorable to tissue restoration. IL-1 also activates several cell types involved in the healing process, such as endothelial cells, which are stimulated to express adhesion molecules and chemotactic proteins. This promotes coordinated efforts in tissue repair by allowing immune cells, fibroblasts and endothelial cells to migrate to the wound bed. Furthermore, IL-1 activates fibroblasts, which are required for the production of the ECM—this serves as a scaffold for tissue regeneration and wound healing [69]. IL-1 promotes the production of collagen, fibronectin, proteoglycans and other ECM components, giving healing tissue tensile strength and promoting granulation tissue development.

The fibroblast growth factor (FGF) family, particularly FGF-7 (keratinocyte growth factor) and FGF-10, play a significant role in fibroblast–keratinocyte interactions. FGFs secreted by fibroblasts bind to FGF receptors on keratinocytes, activating intracellular signaling pathways such as the mitogen-activated protein kinase (MAPK)/ERK pathway. This leads to the regulation of keratinocyte proliferation, migration and differentiation, influencing tissue repair and regeneration processes. Therefore, the release of cytokines from cells within a wound is integral to normal healing, facilitating effective communication and recruitment. It is imperative to consider the various cellular populations involved in the repair of injured skin when designing biomaterials and evaluating their wound-healing potential [70].

Angiopoietin-1 (Ang-1) is a soluble growth factor that has been the subject of extensive research due to its remarkable ability to promote cell adhesion and survival in various cell types, such as cardiomyocytes, endothelial cells and fibroblasts [71, 72]. Interacting with the endothelial-specific Tie2 receptor, Ang-1 exerts a robust regulatory influence over emerging blood vessels, significantly enhancing vascular stability and integrity in mature vessels. Direct cell adhesion to Ang-1 is coordinated by integrins α_5_β_1_ and α_v_β_5_—in fibroblast assays using integrin-blocking agents, Ang-1-mediated cell adhesion was significantly reduced [71, 72]. Ang-1 binds to integrins in a highly specific and selective manner, triggering a cascade of events within the cell that ultimately results in enhanced adhesion and survival. Ang-1 also interacts directly with cardiac and skeletal myocytes in an integrin-dependent manner to activate survival pathways and support their viability under adverse conditions [71, 72]. Ang-1 markedly promotes cardiomyocyte survival under stress and limits ischemia-induced injury. As such, angiopoietin regulation may prove therapeutically valuable in cardiac remodeling and the impedance of heart failure development.

The diabetic phenotype of wound healing is partly characterized by impaired neovascularization and deficient recruitment of endothelial progenitor cells (EPCs). Ang-1 is recognized as a potent mobilizer of EPCs from the bone marrow, and this process is mediated by stem cell factor (SCF) and MMP-9. Using an endothelial lineage labeled murine model of diabetes to track bone marrow-derived EPCs, Balaji *et al*. showed that dorsal wounds in bone marrow transplanted diabetic mice overexpressing Ang-1 had significantly improved re-epithelialization and neovascularization dependent on EPC recruitment from the bone marrow. Moreover, MMP-9^-/-^ mice demonstrated impaired EPC mobilization compared with wild-type controls, which was in turn rescued by SCF administration [73]. Previous studies have explored the effectiveness of Ang-1 gene-modified bone marrow mesenchymal stem cells (Ang1-MSCs) in promoting cutaneous excisional full-thickness wound healing in rats. The use of Ang1-MSCs demonstrated a significant acceleration of wound healing by fostering both epidermal and dermal regeneration, along with enhancing angiogenesis [74]. Most recently, Ang-1 has been shown to enhance angiogenesis synergistically with VEGF in dual gene-activated dermal scaffolds (DGADSs) used to heal deep skin defects. Wang *et al*. designed scaffolds by incorporating nanocomposite particles of polyethylenimine (PEI)/multiple plasmid DNAs encoding VEGF and Ang-1 in a 1:1 ratio. In a rat full-thickness skin defect model, they demonstrated that these DGADSs led to significantly increased fibroblast infiltration, collagen deposition and neovascularization, accompanied by maturation of the vascular network. Hence, DGADSs show promise in facilitating the vascularization of functional tissue-engineered dermal substitutes for applications in wound repair [75].

Despite the considerable potential of Ang-1 in various applications, such as vasculogenesis, cardioprotection and wound regeneration, the intricate and multi-domain structure of the protein has posed challenges for its widespread use as a therapeutic agent. To overcome this limitation, an engineered variant known as Cartilage Oligomeric Matrix Protein-Angiopoietin-1 (COMP-Ang1) has been developed. COMP-Ang1 has demonstrated the ability to activate the Tie2 pathway with several-fold greater potency than Ang-1, both *in vitro* and *in vivo*. This engineered variant has shown beneficial effects on skeletal muscle regeneration, wound healing and angiogenesis, as well as anti-inflammatory effects [76]. Researchers demonstrated that COMP-Ang1 delivery, either via systemic therapy with the adenovirus-encoded gene or by topical delivery of the recombinant protein, accelerated wound closure and epidermal and dermal regeneration, enhanced angiogenesis and lymphangiogenesis and increased blood flow to excisional full-thickness tail wounds in a diabetic mouse model. Another challenge associated with using the entire Ang-1 protein in therapeutic applications is related to the production of recombinant Ang-1 protein, as producing large quantities of biologically active protein can be a complex and expensive process. Moreover, there is a risk of protein denaturation during production and purification, which can render the protein inactive or less effective [72].

## Traditional strategies in wound healing

### Collagen

The ECM in human skin consists of intricate macromolecules, including fibrous components such as collagen and elastin, as well as glycoproteins like fibronectin, proteoglycans and laminins. These molecules play a crucial role in the orchestrated process of wound healing, actively participating in tissue function, growth and repair [77–79]. Collagen, particularly type I, stands out as a predominant structural protein in the skin. Upon injury, collagen triggers platelet activation and aggregation, leading to the deposition of a fibrin clot at the site of injury. Fibroblasts play a key role in collagen deposition, and the subsequent degradation of collagen releases fragments that promote fibroblast proliferation, angiogenesis and re-epithelialization [80, 81]. Given its significant role, collagen proves to be an ideal material for biomaterials designed to target wound healing [82].

**Figure 3.**
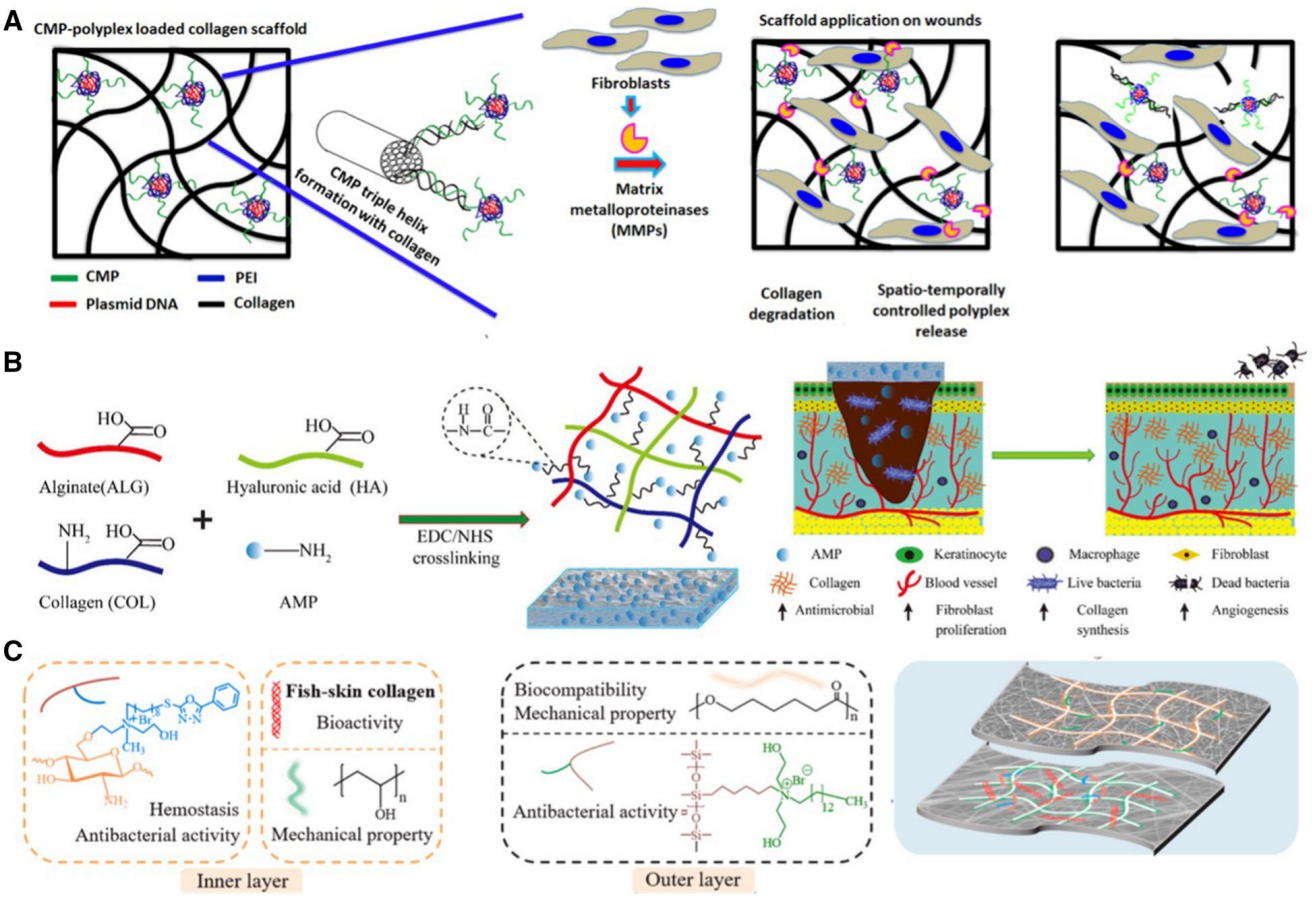
Peptide modification of biomaterials in wound healing. (**A**) Illustration of the CMP-polyplex-loaded co-gel scaffold and its subsequent remodeling following wound fibroblast-mediated collagen degradation and polyplex release, leading to cell-driven growth factor expression and enhanced wound healing. Reproduced with permission from Thapa *et al*. [83]. (**B**) Schematic of the preparation of the ALG/HA/COL-AMP wound dressings. Reproduced with permission from Lin *et al*. [93]. (**C**) Illustration of a PQCQS bilayered electrospun nanofibrous wound dressing evaluated in a rabbit ear full-thickness wound defect model. Reproduced with permission from Gao *et al*. [90].

Collagen, a versatile biomaterial, can be sourced from various animals, including bovine, equine, avian and porcine origins. Its attributes, including biocompatibility, low immunogenicity, the capacity to elicit a pro-healing cellular response, and ease of application in the form of hydrogels, porous structures and fibrous dressings, make collagen successful in demonstrating hemostatic and antibacterial properties, as well as biodegradable characteristics in both *in vitro* and *in vivo* settings. Collagen, in particular, has found application as an adjunct in the therapy of chronic wounds characterized by persistent inflammation, increased destruction of ECM components and improper activation of soluble mediators. These benefits are actively under investigation and refinement for their application in wound healing [60]. For instance, a study by Thapa *et al*. showed that the application of a collagen mimetic peptide (CMP) in CMP-modified PDGF polyplex-loaded co-gel, promoted collagen production, cell proliferation, cell migration and myofibroblast activity (Figure 3A**)** [83].

### Chitosan

Chitosan is a distinctive linear amino polysaccharide derived from the N-deacetylation of chitin, a prevalent biopolymer presents in fungi and the exoskeleton of crustaceans [82]. Chitosan has been shown to have other favorable attributes, including low toxicity, hemostatic properties and antibacterial and biodegradable characteristics [84, 85]. Chitosan has often been used as a complement to collagen to improve collagen’s poor mechanical and thermal properties. In the 1980s, the Damour group conducted tests involving collagen-chitosan-glycosaminoglycans as an artificial dermis in rats, mice and humans [86]. Studies since 1996 have demonstrated that the composites of collagen: chitosan are superior in mechanical stability compared to collagen alone [87]. For instance, the incorporation of chitosan in collagen-based wound dressings has been found to enhance their antibacterial properties and promote faster healing [88]. Furthermore, a study by Bakhsheshi-Rad *et al*. showed that using chitosan as a coating for implants has improved their biocompatibility and reduced the risk of infection [89]. A study by Gao *et al*. demonstrated the hydrophilic, biocompatible and hemostatic properties of the inner membrane in their bilayered nanofibrous membrane (PQCQS) for wound-healing applications (Figure 3B) [90]. This inner membrane was composed of polyvinyl alcohol, collagen and quaternized chitosan.

### Peptide-based materials

Addressing microbial infection through the inclusion of antimicrobial peptides (AMPs) is a significant development in wound healing [91]. As of now, a multitude of studies have indicated that AMPs effectively combat infection, thereby contributing to the improvement of the wound-healing process [92]. For instance, the ALG/HA/COL-AMP antimicrobial dressings enhance re-epithelialization, collagen deposition and angiogenesis (Figure 3C) [93]. AMPs are evolutionarily conserved products of the innate immune system [94]. Despite considerable structural variability and ranging from 10 to 50 amino acids in length, most AMPs share a cationic and amphipathic structure [95]. The mechanism underlying their antimicrobial action primarily involves electrostatic interaction with anionic phospholipids present in the microbial cell membrane [96]. The distinctive mode of action exhibited by AMPs sets them apart from conventional antibiotics, which often rely on single enzymatic mechanisms and are more susceptible to inducing resistance [97]. For instance, the peptide A3-APO demonstrates potent antimicrobial action by disrupting the integrity of microbial cell membranes, surpassing the effectiveness of standard antibiotics and providing an efficacious treatment option for experimental multidrug-resistant *A. baumannii* wound infections after burn injuries [98]. In addition, the capacity of the peptide A3-APO to penetrate the microbial cell membrane and access the cytoplasm enables the targeting of diverse intracellular components, including DNA, RNA, proteins and enzymes.

Several peptides not only activate the release of cytokines and growth factors but also stimulate the recruitment of neutrophils and macrophages [99, 100]. For example, the efficacy of HB-107 was demonstrated when it was suspended in a hydrogel of carboxymethyl cellulose in a full-thickness murine wound-healing model, increasing leukocyte infiltration and keratinocyte hyperplasia as well as effectively accelerating wound healing by stimulating IL-8 secretion from cultured endothelial cells [100]. Tylotin, in particular, has been found to directly enhance the motility and proliferation of fibroblasts, keratinocytes and vascular endothelial cells while promoting the release of TGF-β1 and IL-6 [101]. Similarly, the SR-0379 peptide has shown the capability to enhance the proliferation of normal human dermal fibroblast cells (NHDFs) through the PI3 kinase-Akt-mTOR pathway via integrin-mediated interactions and direct tube formation of endothelial cells and NHDFs in addition to its antimicrobial activity [102]. Some researchers treated skin wounds established in mice using peptide Ser-Ile-Lys-Val-Ala-Val (SIKVAV)-modified chitosan hydrogels, observing enhanced collagen fiber deposition and secretion of EGF, bFGF, VEGF and TGF-β1, which enhanced re-epithelialization in the skin wound [103–105].

Certain peptides have been observed to interact with the host immune system and modulate human macrophage differentiation, leading to the development of a pro-healing phenotype. For example, synthetic, innate defense regulator (IDR) peptides such as IDR-1018 are designed based on natural host defense peptides and drive macrophage differentiation toward an intermediate M1-M2 state. As such, they can enhance anti-inflammatory functions while maintaining certain pro-inflammatory processes required to resolve infection [106]. AMPs can also regulate the inflammatory response by promoting the release of anti-inflammatory cytokines while suppressing pro-inflammatory cytokines, thereby resolving inflammation and promoting healing. The cationic AMP LL-37, a proteolytic fragment of the cathelicidin family, triggers the formation of NETs, which contribute to defense against pathogens and chronic injury [107]. Similarly, LL-31 possesses both antimicrobial and immunomodulatory properties, which accelerate the recruitment of neutrophils and monocytes to the site of infection and reduce the production of pro-inflammatory cytokines such as IL-8 and IL-6 while increasing the secretion of anti-inflammatory cytokines such as IL-10 [108, 109]. PR-39, found in pig leukocytes, exhibits antimicrobial and immunomodulatory activities by enhancing the recruitment and activation of neutrophils and macrophages, leading to increased phagocytosis and antimicrobial responses [110]. In addition, PR-39 can downregulate the production of pro-inflammatory cytokines such as IL-1β and TNF-α while promoting the release of anti-inflammatory cytokines like IL-10 [111].

The adoption of peptide-based materials is contingent on enhancing peptide proteolytic stability and spatiotemporal presentation to enable long-term and tissue-specific biomaterial applications. Another challenge is to effectively synchronize the biodegradability of peptide-based materials with concurrent neotissue formation without compromising their mechanical properties [112]. Ideally, one would achieve a peptide degradation profile that is controlled by signaling molecules generated during the wound-healing process. For example, Schultz *et al*. reported an hMSCs-laden PEG hydrogel cross-linked with an MMP-sensitive peptide through the incorporation of MMP binding and cleavage sequences. This hydrogel scaffold could be degraded proteolytically following MMP secretion by encapsulated cells, simultaneously promoting hMSCs migration and matrix remodeling [113]. Although peptide-based materials allow for control over cell recruitment and downstream cytokine release, an in-depth mechanistic exploration of each material is required. Peptides are often highly ubiquitous and lack specificity to particular cell types and signaling pathways, and the provoked downstream signaling may also differ from the response induced by the native protein [114]. This complexity makes it difficult to produce and modulate the desired response for a specific regenerative biomaterial and avoid unintended *in vivo* toxicity or side effect profiles. Finally, the physical and chemical properties of these materials must be optimized when it comes to the functional and structural reproducibility of controlled peptide release, sterilization techniques and implantation methods that ensure tissue adhesion and incorporation, all of which are necessary before proceeding with clinical translation [115].

### Angiopoietin-1-derived QHREDGS peptide hydrogel: a case study for biomimetic material development

Within the Ang-1 protein, there is a specific sequence of only seven amino acids (QHREDGS) known as Q-Peptide. This short sequence has been identified as the integrin-binding sequence of Ang-1 and, as a synthetic peptide, is just as effective as the full Ang-1 protein in promoting cell adhesion and survival. This discovery has opened up new possibilities in the field of regenerative medicine and tissue engineering [116, 117]. One of the key advantages of Q-Peptide is its ease of production. Unlike the production of full-length proteins, synthesizing small peptides like Q-Peptide is a relatively straightforward process. Additionally, Q-Peptide can be easily conjugated to various materials, making it versatile for use in different applications. For example, QHREDGS-immobilized surfaces induced integrin-mediated osteoblast differentiation and produced significantly more bone matrix deposition and mineralization, processes that are both critical to bone regeneration [118].

In the context of myocardial infarction (MI), researchers have explored using Q-Peptide to augment hydrogels used for biomaterial-delivered cell therapies. Cardiomyocytes encapsulated in chitosan hydrogels conjugated with immobilized Q-Peptide and mixed with collagen I showed improved morphology, survival and metabolic activity, as well as a greater success rate of obtaining a beating construct [119]. Furthermore, when QHREDGS is conjugated to this collagen-chitosan hydrogel, it enhances endothelial cell survival and stimulates these encapsulated cells to form tube-like structures [116]. In experiments where integrins α5β3 or α5β1 were blocked with antibodies during cell encapsulation in peptide-modified hydrogels, the process of tube formation was halted [116]. This suggests that these specific integrin subtypes are essential for Q-Peptide’s beneficial effects. Understanding the integrin receptors involved provides valuable insights into the mechanisms of action of Q-Peptide in MI and other related conditions. For example, QHREDGS-conjugated hydrogels injected into the peri-infarct zone in a rat MI model resulted in significantly more cardiomyocytes in the MI zone, which were shown to interact with Q-Peptide via β1-integrins [120]. Remarkably, these QHREDGS-conjugated hydrogels attenuated post-MI cardiac remodeling and induced significant cardiac functional and morphological improvements in comparison to controls [120].

Recent studies have extended the scope of Q-Peptide’s applications to wound healing, particularly through its effects on instrumental cell types such as macrophages and fibroblasts. In an example, fibroblast monolayers seeded on Q-Peptide conjugated collagen-chitosan hydrogels uniquely secreted both pro- and anti-inflammatory cytokines, as well as anti-fibrotic cytokines and those required for ECM deposition [121]. These fibroblasts also demonstrated immunomodulatory effects on macrophages and up-regulated the expression of critical wound-healing genes. In diabetic models, Q-Peptide has shown promise both *in vitro* and *in vivo*. When normal and diabetic human primary keratinocytes were exposed to Q-Peptide immobilized on chitosan-collagen films in an *in vitro* setting, they exhibited enhanced attachment, survival and collective migration [122]. Furthermore, in full-thickness excisional wounds in a diabetic mouse model, the application of an immobilized Q-Peptide chitosan-collagen hydrogel accelerated wound closure when compared to a commonly used porous collagen dressing [122], resulting primarily from enhanced re-epithelialization and increased granulation tissue formation. that the topical application of QHREDGS peptide-modified hydrogels expedited wound closure. This acceleration was attributed to the enhancement of re-epithelialization through increased migration and survival of human keratinocytes [123]. These studies suggest that Q-Peptide could potentially address one of the key challenges in diabetic wound healing: a lack of epithelial cell migration and delayed re-epithelialization.

It is worth noting that Q-Peptide has demonstrated distinct efficacy differences between its soluble and immobilized forms. Previous studies suggest that when Q-Peptide is immobilized, it becomes more effective compared to its soluble counterpart [116, 124]. This enhanced effectiveness is particularly evident in promoting tube formation and cell survival [116]. These findings underscore the significance of the material context in which Q-Peptide is presented to cells. For instance, the soluble form showed minimal cytokine release, indicating limited activation of macrophages [125]. In contrast, the immobilized peptide induced a more pronounced and balanced immune response. This distinction is vital because an orchestrated immune response is crucial for effective wound healing. Moreover, findings from both *in vitro* and *in vivo* studies involving diabetic subjects indicate that although the soluble peptide offers a degree of protection against oxidative stress, it does not influence the process of re-epithelialization as significantly as the peptide hydrogel in its conjugated form [122].

## Modulating innate immunity in the new generation of wound-healing biomaterials

In the intricate process of wound healing, biomaterials play a pivotal role in orchestrating the delicate balance between pro-inflammatory and anti-inflammatory responses through the modulation of specific signaling pathways. Inflammatory response is crucial for the initiation of the wound-healing process, angiogenesis and the elimination of pathogens from the wound bed. If the inflammation is to be completely abolished, wound healing cannot take place. Yet, persistent inflammation leads to non-healing wounds and complications in the wound-healing process. Ideally, an inflammatory response will initiate the cascade of wound-healing events and rapidly resolve to let regenerative processes take place. Biomaterials play a significant role in modulating these responses, and understanding their interactions is key to developing effective wound-healing strategies.

During the initial stages of wound healing, pro-inflammatory cells, such as neutrophils and macrophages, recruited to the site of the injury can help in the clearance of debris, dead cells and pathogens, creating an optimal microenvironment for the healing process to commence. In this case, some biomaterials induce pro-inflammatory reactions by activating pathways, such as NF-κB [126], MAPK [127] and Janus kinase/signal transducer and activator of transcription (JAK/STAT) [128], leading to the release of pro-inflammatory cytokines, such as TNF-α [129], IL-6 [130], IL-1β [129], CXCL2, MMPs [131], CXCL8 [132] and monocyte chemoattractant protein-1 as well as the recruitment of immune cells crucial for early-stage processes like debris clearance and infection prevention. As Tamada *et al*. reported, fibroblasts, cultured on silk fibroin films and sponges, exhibited up-regulated expressions of IL-1β and TGF-β related to wound repair compared to their collagen counterparts [129]. Fibroblasts exhibit a remarkable sensitivity to the influence of TGF-β, a molecule predominantly secreted by wound-associated pro-repair macrophages, which facilitate collagen production and provide mechanical strength to the wound. In another example, the combined fibroin/aloe gel wound dressing, developed from natural extracts, promotes skin wound healing by enhancing VEGF secretion linked to the activation of the MAPK signaling pathway [127]. These findings suggest that biomaterials have the potential to enhance pro-inflammatory cytokines and cells, thereby accelerating wound healing and tissue reconstruction, making them promising candidates for functional wound dressings.

On the other hand, anti-inflammatory reactions are also integral to the wound-healing process, working in a cycle with pro-inflammatory responses to maintain a balanced and controlled environment. Certain biomaterials are crafted to foster an anti-inflammatory environment by engaging with pathways like TGF-β/Smad [133], peroxisome proliferator-activated receptor-γ (PPAR-γ) [134], Janus kinase- signal transducers activators of transcription (JAK-STAT) [126], promoting the release of anti-inflammatory cytokines, such as IL-4 [135], IL-10 [136] and IL-13 [137], and facilitating the transition to tissue repair. For example, the incorporation of curcumin nanoparticles into collagen-chitosan scaffolds significantly enhances cutaneous wound healing in a rat model, as evidenced by accelerated vessel formation, greater granulation tissue density and enhanced collagen content, with a simultaneous regulation of TGF-β1 and Smad7 mRNA expression, emphasizing the anti-inflammatory and regulatory effects of the biomaterials on the wound-healing process [133]. A novel double network hydrogel composed of squid cartilage type II gelatin and hyaluronic acid (HA) exhibits anti-inflammatory properties by effectively preventing the resting state macrophage from transitioning (M0) into a pro-inflammatory state (M1) by inhibiting the Jun N-terminal kinases (JNK) pathway [134]. Simultaneously, it facilitates M2 polarization through the activation of the STAT6/PPAR-γ pathway. This dual action results in a substantial reduction in levels of TNF-α, IL-1β, IL-6 and iNOS, accompanied by a significant increase in TGF-β levels.

However, excessive suppression of pro-inflammatory responses by biomaterials may accidentally hinder the natural healing cascade, potentially delaying wound repair. Pro-inflammatory responses are essential for combating pathogens and preventing infections at the wound site [137]. Excessive suppression may compromise the immune system’s ability to defend against invading microorganisms, resulting in persistent infections that hinder the healing process [138]. In addition, suppressing pro-inflammatory signals excessively may hinder the activation of macrophages, fibroblasts and angiogenesis, leading to the disturbance of matrix synthesis [129], inadequate blood supply [127] and affecting the structural integrity of the healed tissue [60]. In summary, achieving the optimal and dynamic balance in biomaterial design is an ongoing challenge, with researchers exploring strategies such as incorporating anti-inflammatory agents, manipulating surface properties, and fine-tuning release kinetics to guide the immune response effectively through the phases of wound healing.

### Biomaterials: targeting neutrophils

The significance of neutrophils (NEUs) in the process of integrating biomaterials into wound healing cannot be overstated. NEUs serve as the initial defense against implanted materials, exerting considerable influence over the fate of these materials. They achieve this through the secretion of chemical substances and the formation of NETs. How NEUs respond is highly dependent on signals from the biomaterial and its immediate environment, which sets the stage for subsequent immune reactions. NEUs may either act as facilitators of tissue repair or disruptors of the healing process by releasing inflammatory signals and generating excessive NETs [139, 140].

**Figure 4.**
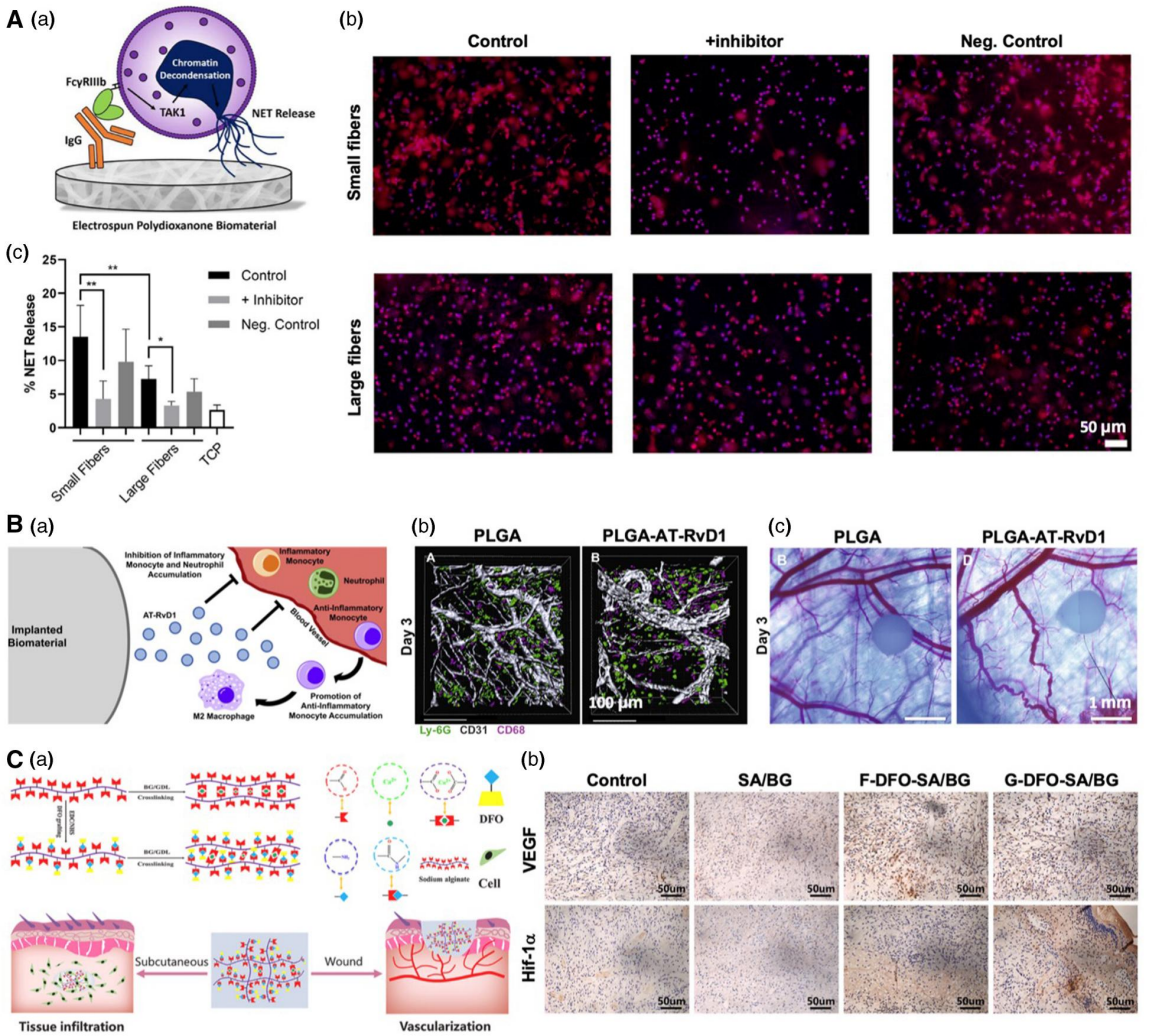
Biomaterials targeting neutrophils. (**A**) (a) The schematic mechanism of human neutrophil FcγRIIIb regulating neutrophil extracellular trap release in response to electrospun polydioxanone biomaterials. (b) Fluorescent micrographs of neutrophils on the electrospun biomaterials at 3 h after seeding. NETs are shown in red and nuclei in purple. The scale bar is 50 µm. (c) The quantification of percent NET release (*n* = 6) indicates that inhibition of TAKI reduces NET formation on both the small and large fiber electrospun biomaterials. Reproduced with permission Fetz *et al*. [148]. (**B**) (a) The schematic figure showing aspirin-triggered resolvin D1-modified materials promote the accumulation of monocyte and neutrophil and enhance vascular remodeling. (b) Local delivery of at-RvD1 increases CD68+ monocytes and macrophages. Imaris renderings of CD68+ macrophage and Ly-6G+ neutrophil accumulation. (c) Delivery of at-RvD1 promotes vascular remodeling. Reproduced with permission from Sok *et al*. [162]. (**C**) (a) The mechanism of modulating degradation of sodium alginate/bioglass hydrogel for improving tissue infiltration and promoting wound healing. (b) Immunohistochemistry staining of VEGF and Hif-1α. Reproduced with permission from Zhang *et al*. [170].

The design of biomaterials significantly governs NEU activity. NEU recruitment and activation are closely tied to the concentration of proteins adhered to the biomaterial’s surface. This concentration is intricately linked to the biomaterial’s biocompatibility and surface area. Non-porous biomaterials with a low surface area-to-volume ratio have often been linked to prolonged inflammation. The presence of nanofibrous networks, nanoparticles and micro-pores significantly influences interactions between NEUs and macrophages, angiogenesis and overall tissue regeneration [141]. Naturally derived polymers, which resemble the fibrous ECM, hold great promise in maintaining material functionality and fostering positive inflammatory responses [142]. These responses ultimately lead to tissue regeneration without fibrosis. Chitosan scaffolds, for instance, have been shown to enhance the presence of neutrophils and expedite the recovery of chronic wounds [143–145]. Fibronectin-based hydrogels can also reduce the neutrophil count and accelerate wound healing [146]. Fabrication of fibrous biomaterials such as polydioxanone through an electrospinning process is another way to minimize NET extrusion [140, 147–149]. Specifically, the inhibition of TAK1 reduces biomaterial-induced NET release (Figure 4A) [148]. Incorporation of silver and gold particles or carbon nanotubes into the structure of naturally derived hydrogels is another effective way to regulate the number of attached neutrophils and accelerate wound healing [150–152].

The manipulation of biomaterial properties is a vital strategy for controlling the adhesion and activation of neutrophils when these materials are implanted. Studies have indicated that non-ionic hydrophilic surfaces can reduce the production of pro-inflammatory cytokines, indicating their potential to limit both leukocyte adhesion and macrophage fusion and promote regenerative inflammation [153]. Biomaterials such as poly(ethylene glycol) (PEG), HA, glycosaminoglycans, chitosan, heparin and phosphatidylcholine have been used to increase hydrophilicity and reduce protein adsorption through various processes [154–158]. Synthetic polymers such as poly(lactic acid), poly(lactic-co-glycolic acid) (PLGA) and poly(vinyl alcohol) have also shown potential for enhancing biocompatibility by limiting protein adsorption and consequently reducing NEU recruitment [159–161]. For instance, Sok *et al*. conducted a study where they encapsulated aspirin-triggered resolvin D1 (AT-RvD1) within a PLGA scaffold, aiming to explore how this treatment affected the behavior of neutrophils, macrophages and vascular remodeling (Figure 4B) [162]. In a murine dorsal skinfold window chamber model, they observed that AT-RvD1 treatment led to a decrease in both the presence and migration of neutrophils. Additionally, wounds treated with PLGA-AT-RvD1 showed an increase in CD49+ neutrophils, which play a role in vascular remodeling, along with a lower ratio of neutrophils to monocytes/macrophages, indicating a reduction in inflammation. It has also been shown that biomaterials with neutral surface charges can minimize protein adsorption, resulting in low NEU activation. Surface charge modification through grafting zwitterionic ligands and PEG has been suggested as an effective way to mitigate electrostatic interactions [163–166].

Material degradability is another essential aspect affecting tissue regeneration and biomaterial integration. Biomaterial degradation can alter surface topography and charge, affecting protein adsorption and NEU activation. Moreover, degradation products may be toxic, resulting in inflammation in the vicinity of the implant and the attraction of more NEUs [167, 168]. To mitigate immunological responses and facilitate tissue regeneration, it is preferred to use polymers with a slow degradation rate whose degradation products are bioresorbable. Natural polymers typically yield more bioresorbable degradation products compared to synthetic materials, making them suitable choices for biomaterials. However, they tend to degrade rapidly, potentially leading to immune reactions. A hybrid approach that combines natural and synthetic polymers can allow precise control over degradation rates and mechanical properties such as elasticity and strength [169, 170]. For instance, grafting deferoxamine onto SA/BG hydrogel speeds up the degradation, which results in better tissue penetration (Figure 4C) [170].

### Biomaterials: targeting macrophages

**Figure 5.**
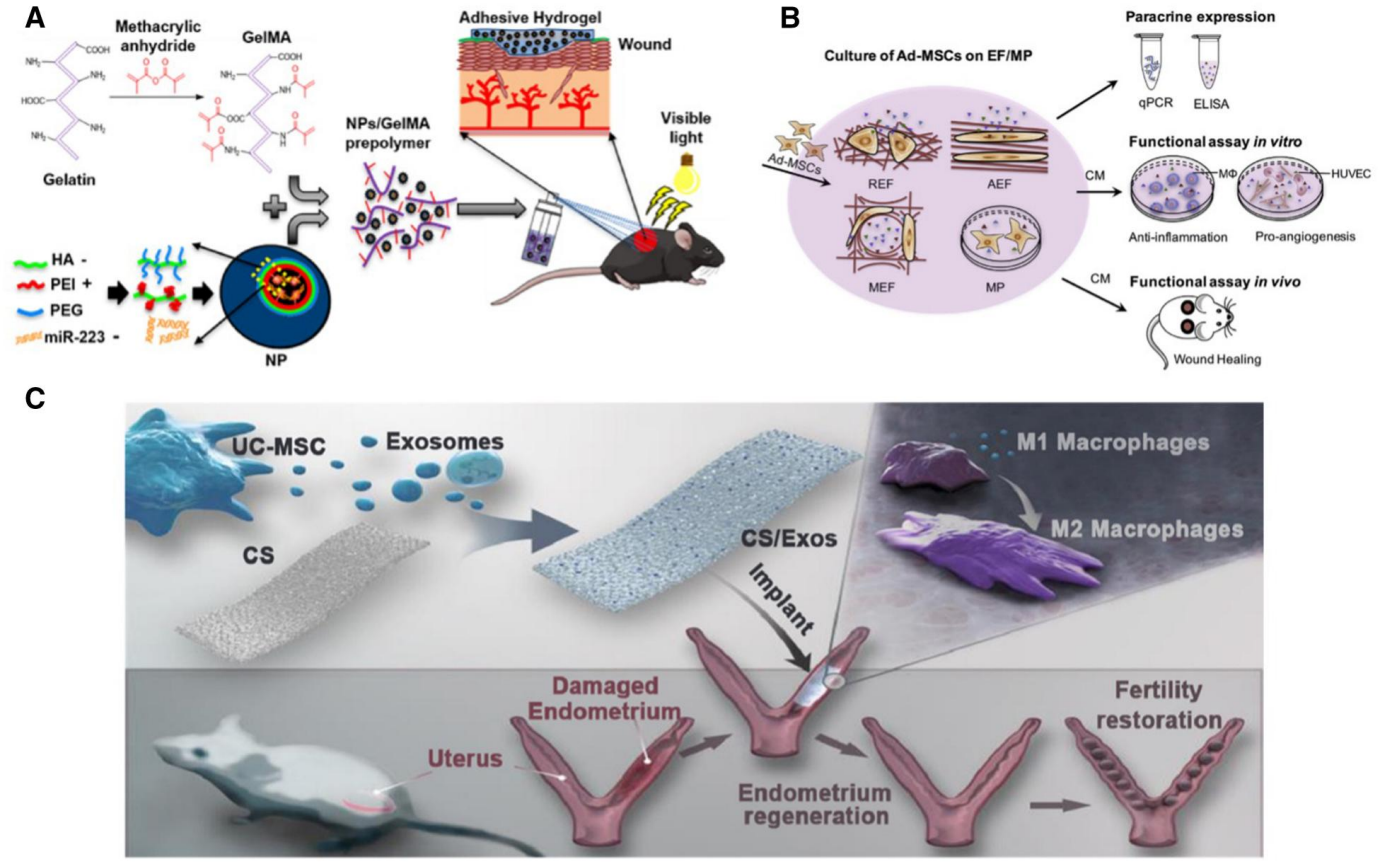
Biomaterials targeting macrophages. (**A**) Schematic illustration of the process for the formation of NP/miR-223*-laden GelMA hydrogels and the use of these adhesive hydrogels for wound healing. Reproduced with permission from Saleh *et al*. [173]. (**B**) Experimental design for investigating the influence of fiber morphology and orientation on the paracrine secretion and function of Ad-MSCs. The scaffolds for cell culture included electrospun fibers in random, aligned and mesh organization, which were designated as random electrospun fibers (REF), aligned electrospun fibers (AEF) and mesh-like fiber (MEF), respectively. The cultures on EFs were compared to Ad-MSCs cultured on a polystyrene microplate (MP). Reproduced with permission from Su *et al*. [179]. (**C**) Schematic illustration of an exosome-functionalized, immunomodulatory collagen porous scaffold (CS/Exos) for endometrium regeneration and fertility restoration. Reproduced with permission from Xin *et al*. [185].

As the dynamic role of macrophages is becoming increasingly recognized as crucial in wound healing, some recent studies have explored the use of therapeutic biomaterials to promote cutaneous wound healing by modulating macrophage polarization from an inflammatory (M1-like) phenotype to that of pro-healing (M2-like) phenotype. For example, Zhu *et al*. emphasized the significance of M2 macrophage polarization in wound closure rate and collagen deposition by comparing normal mice and macrophage-depleted mice [171]. The application of nanoparticles to modulate macrophage polarization and improve wound healing has also been investigated. For example, the treatment of healthy and diabetic mice with Konjac glucomannan-modified SiO_2_ nanoparticles accelerated wound closure and promoted the phenotypic transition of macrophages from M1 to M2. The mechanism for this transition likely involved the formation of mannose receptor nanoclusters on the surface of macrophages [138]. In another study, You *et al*. loaded silver nanoparticles into a collagen-chitosan scaffold (NAg-CSS). Along with its antimicrobial effects, treatment with NAg-CSS resulted in a strong anti-inflammatory response, reducing the infiltration of inflammatory cells and CD68 expression [172]. Moreover, the miR-223 5p mimic was encapsulated in HA-based nanoparticles, which were then loaded into a GelMA-based hydrogel (Figure 5A). Faster wound closure and thicker collagen layers were observed, along with an increase in Arg1/iNOS2 ratio, indicative of an M1 to M2 shift in the macrophage phenotype [173].

Peptides such as SIKVAV and QHREDGS, derived from laminin and angiopoietin-1, respectively, have also been incorporated into hydrogels and shown to possess wound-healing immunomodulatory properties [174]. Specifically, the peptide-hydrogel system elicited a unique macrophage polarization, with increased secretion of both pro-inflammatory and anti-inflammatory cytokines [175]. Hydrogels loaded with growth factors have also been shown to modulate macrophage phenotypes and promote wound healing. For example, Chen *et al*. encapsulated VEGF inside a four-armed benzaldehyde-terminated polyethylene glycol and dodecyl-modified chitosan hybrid hydrogel. A higher M2:M1 macrophage ratio was observed, along with a high degree of hemostatic capability and increases in wound closure, blood vessel density, and collagen deposition [176]. Other bioactive agents, including JK1, a pH controllable H_2_S donor and prostaglandin E2 [177, 178] have also been delivered using hydrogels and demonstrated their potential in modulating macrophage polarization in the context of wound healing.

Stem cells have been gaining attention in recent years for their incorporation within matrices to induce macrophage phenotype modulation. Su *et al*. seeded adipose-derived mesenchymal stem cells on polycaprolactone electrospun fibers with different topographies (Figure 5B). It was observed that a mesh-like fiber structure was optimal, as it enhanced the expression of IL-10 and Arg-1 (both of which are macrophage-secreted anti-inflammatory factors) in addition to a faster wound closure rate and improved collagen organization [179]. In another study, bone marrow-derived mesenchymal stem cells were seeded on a hybrid hydrogel composed of unsaturated arginine-based poly(ester amide) and glycidyl methacrylate chitosan (MSC-seeded ACgels). In addition to promoting wound closure, re-epithelialization and vascularization, MSC-seeded ACgels increased the presence of M2-like macrophages, and a greater portion of these macrophages expressed IL-10. Moreover, a reduction in M1-like macrophages was observed, and fewer of these macrophages expressed TNF-α. Cell sheets from cultured stem cells have also been explored as a therapeutic wound-healing biomaterial to elicit a desired macrophage response. Yang *et al*. demonstrated that curcumin-induced bone marrow-derived mesenchymal stem cell sheets reduced the presence of M1 macrophages and increased Relma and Arg1 in a full-thickness wound model, both of which are associated with M2 macrophages [180]. Additionally, metal ions such as Co^2+^ have also been shown to shift macrophages to a pro-healing phenotype *in vivo*, and it was observed that CD208+ macrophages increased with the application of a gauze and sodium alginate composite dressing containing Co^2+^ [181]. Poly(methacrylic acid-co-methyl methacrylate) beads have also been explored as a wound-healing biomaterial that modulates macrophage polarization and promotes vascular regeneration [182].

Wound-healing biomaterials that modulate macrophage polarization have also been explored in other epithelial tissues, such as abdominal tissue. Darzi *et al*. investigated the use of a polyamide and gelatin mesh (PA + G) seeded with human endometrial mesenchymal stem cells (eMSCs) to repair abdominal subcutaneous wounds in immunocompetent and immunocompromised mice, with the objective of treating pelvic organ prolapse. In the immunocompromised mice, it was found that eMSCs/PA + G treatment increased the presence of M2 macrophages a few days following implantation [183]. In another study, the healing of abdominal wounds in mice improved through the application of a chitosan-HA (CS/HA) hydrogel, resulting in greater granulation tissue thickness, vascular structure area and collagen deposition. It was also observed that the M2 macrophage population was significantly higher in wounds treated with the CS/HA hydrogel [184]. It has also been reported that the delivery of umbilical cord mesenchymal stem cell (UC-MSC)-derived exosomes using a collagen scaffold (CS/Exos) promoted endometrium regeneration (Figure 5C). In addition to a thicker repaired endometrium, increased capillary formation, and reduced endometrial fibrosis, CS/Exos-treated wounds had high numbers of CD163^+^ cells. Further analysis revealed that the miRNA miR-223-3p in UC-MSC-derived exosomes could be a key player in M2 macrophage polarization by inhibiting stathmin 1 targets [185].

### Biomaterials targeting adaptive immune cells

**Figure 6.**
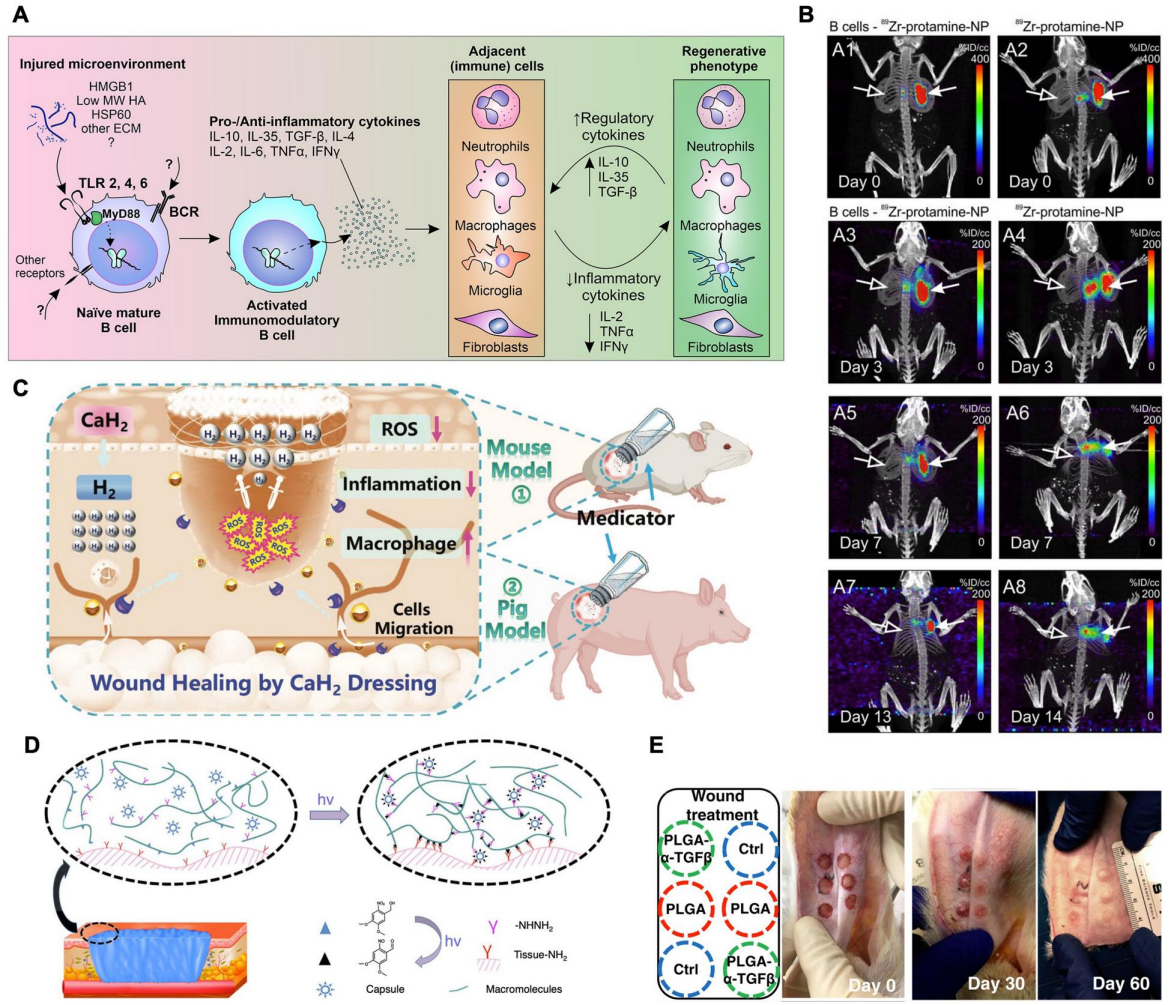
Biomaterials targeting adaptive immune cells. (**A**) B cells applied exogenously into injured tissue microenvironments undergo a regulatory phenotypic shift, altering the response of adjacent resident and infiltrating immune cells to initiate a regenerative cellular and molecular Cascade. Reproduced with permission from Sîrbulescu *et al*. [192]. (**B**) PET/CT tracking of B cells after application to the wound bed. Reproduced with permission Sîrbulescu *et al*. [193]. (**C**) The preparation of CaH_2_ dressing and their application for anti-inflammation and skin wound healing. Reproduced with permission Gong *et al*. [198]. (**D**) Schematic illustration of photo-induced crosslinking mechanism for tissue integration of hydrogel and PLGA-NB capsules and (**E**) representative images of rabbit ear skin wound healing and scar formation in differently treated groups. Left upper panel shows the respective treatment for each wound on the ear. Reproduced with permission Zhang *et al*. [200].

The adaptive immune system, comprising primarily T and B lymphocytes, plays a critical role in wound healing. While the innate immune response acts immediately upon injury, the adaptive immune system provides a more targeted and long-lasting response, essential for resolving inflammation, promoting tissue repair and preventing infection [186]. Recent advances in immunology and biomaterials science have shed light on how the adaptive immune response influences wound healing and how it can be modulated to enhance therapeutic outcomes [187]. T cells, especially CD4+ helper T cells and CD8+ cytotoxic T cells, are vital in the wound-healing process due to their ability to direct the activities of other immune cells, release specific cytokines and modulate various phases of repair. CD4+ T cells secrete various cytokines that can influence the behavior of macrophages, fibroblasts and endothelial cells [188]. Specifically, T-helper type 1 (Th1) cells promote inflammation through the release of IFN-γ, while T-helper type 2 (Th2) cells facilitate wound repair by producing IL-4 and IL-13, which support collagen synthesis and fibroblast activity [189, 190]. Regulatory T cells (Tregs) can also effectively resolve inflammation and promote tissue repair by secreting anti-inflammatory cytokines such as IL-10 and TGF-β [191]. B cells, traditionally known for their role in antibody production, also contribute to wound healing through cytokine secretion and antibody-mediated mechanisms. In the context of wound healing, B cells can influence the inflammatory response and facilitate the clearance of pathogens [192] (Figure 6A). Additionally, B cells participate in the resolution phase by producing growth factors and cytokines that support tissue repair [193] (Figure 6B).

The integration of adaptive immune mechanisms into the design of biomaterials for wound healing represents a frontier in regenerative medicine. By harnessing the specificity and longevity of the adaptive immune response, novel therapeutic strategies can be developed to address the complexities of wound healing, particularly in chronic wounds where traditional treatments fail. Strategies for modulating adaptive immune responses in wound healing encompass a range of innovative approaches aimed at enhancing the repair process through precise control over immune activities. One such strategy involves the use of biomaterials engineered for cytokine delivery. These biomaterials are designed to release specific cytokines that modulate the responses of T and B cells, including IL-10, TGF-β and IL-4 [194, 195]. By promoting a shift toward a pro-healing immune environment, controlled release systems ensure these cytokines are delivered sustainably to the wound site, thereby enhancing the healing process. Yu *et al*. created a hydrogel composed of chitosan and poly[2-(methacryloyloxy)ethyl] trimethyl ammonium chloride [196]. This hydrogel promotes a shift from pro-inflammatory T-helper type 17 (Th17) cells toward anti-inflammatory Treg cells. Such a shift aids in the polarization of macrophages toward an anti-inflammatory state, thereby reducing inflammation. In another study, researchers developed a biodegradable cytogel that incorporates IL-33 into a physically cross-linked DNA hydrogel [197]. This design ensures a continuous local release of IL-33, which facilitates the gathering of group 2 innate lymphoid cells and Tregs. This process aids in preventing chronic inflammation in the context of delayed diabetic wound healing. Gong *et al*. created a CaH_2_ pulvis dressing capable of suppressing the secretion of pro-inflammatory cytokines and promoting the infiltration of Treg cells [198] (Figure 6C). The incorporation of checkpoint inhibitors and co-stimulatory molecules into biomaterials represents another strategy to modulate T-cell activation and function. This method is especially beneficial in wounds where an excessive immune response hinders the healing process. By fine-tuning T-cell activity, these biomaterials can alleviate overly aggressive immune responses and foster a more conducive environment for wound repair. For example, incorporating programmed death-ligand 1 (PD-L1) into biomaterials can engage with the PD-1 receptor on T cells, effectively dampening their activity to reduce inflammation and promote tissue repair in chronic wounds. Su *et al*. designed a thermoresponsive PF-127 hydrogel that gels at body temperature and steadily releases exosomal PD-L1, which binds PD-1 on T cells, reducing T-cell activation [199]. Another advancement involves a wound dressing that employs a photo-crosslinking strategy and microcapsules for the targeted release of a TGF-β inhibitor. This approach reduced the number of CD4+ T cells and minimized scarring in both murine models and larger animal studies [200] (Figure 6D).

## Outlook and future studies

In this review, we have explored the critical role of skin as a protective barrier and the significant economic burden associated with non-healing wounds, scars and burns. Understanding the nuanced mechanisms underlying wound healing, particularly the pivotal role of immunomodulation, is imperative for developing effective therapeutic strategies. The intricate balance between promoting wound closure and minimizing scar formation remains a challenge, necessitating advancements in biomaterial development.

In the investigation of immune-regulated wound healing, several biomaterials, such as drug-laden collagen and chitosan patches, peptide-based hydrogels, and growth factor-laden dressings, have emerged as pivotal players in clinical applications, demonstrating the remarkable potential to modulate the intricate interplay between the immune system and tissue repair processes. Antimicrobial dressings, such as silver ion and antibiotic dressing, reduce the risk of infection, while dressings containing growth factors stimulate cell proliferation, angiogenesis, and tissue repair. Collagen and silicone patches provide structural support, aiding in the formation of connective tissue at the wound edges and mitigating scar formation [82]. These biomaterials often leverage advanced engineering strategies to regulate the release of immunomodulatory factors, cytokines and growth factors, orchestrating a finely tuned immune cascade. Such clinical interventions mark a significant stride toward personalized and effective wound care, providing clinicians with tools to harness the body’s immune mechanisms for accelerated and well-coordinated tissue regeneration. As these innovative biomaterials continue to evolve, their integration into mainstream clinical practices holds promise for revolutionizing wound management and enhancing patient outcomes.

Recent progress in leveraging biomaterials for immunomodulation shows promise in mitigating persistent inflammation, reducing bacterial burden and managing infections. Precisely tailored immune responses can be achieved through the design of biomaterials with controlled release of anti-inflammatory compounds, inherent antimicrobial properties, nanomaterial penetration and bioresorbable capabilities. These innovative approaches, coupled with a deep comprehension of wound biology, hold the potential to redefine wound care and ultimately lead to improved outcomes for patients with both acute and chronic wounds. There can also be inspiration from immunomodulatory biomaterials that promote the regeneration of non-epithelial tissue, including muscle tissue, when designing effective biomaterials for wound-healing applications. For instance, there have been studies on biomaterials that modulate macrophage polarization during the repair of myocardial tissue. There have also been studies on the immunomodulatory properties of biomaterials that target smooth muscle repair.

The incorporation of new techniques to enhance cell-based therapies may also help to achieve the translational advancements required for their widespread adoption into clinical practice. For example, gene editing using the CRISPR/Cas9 approach has also recently been used to precisely edit dendritic cells and preserve an immature cell state with strong pro-angiogenic and regenerative capacity—these cells show enhanced therapeutic potential for accelerating the healing of chronic wounds [201]. Focusing on the role of mechanical forces that shift fibroblasts toward pro-fibrotic phenotypes, leading to myofibroblast differentiation and excessive collagen production, may also prove effective if one can disrupt this mechanical signaling [202]. Finally, as dermal fibroblasts exhibit considerable heterogeneity in response to injury, defining lineage origins of reparative fibroblasts using fate mapping and single-cell RNA sequencing can reveal unique signatures that optimize wound-healing outcomes [203].

**Figure 7.**
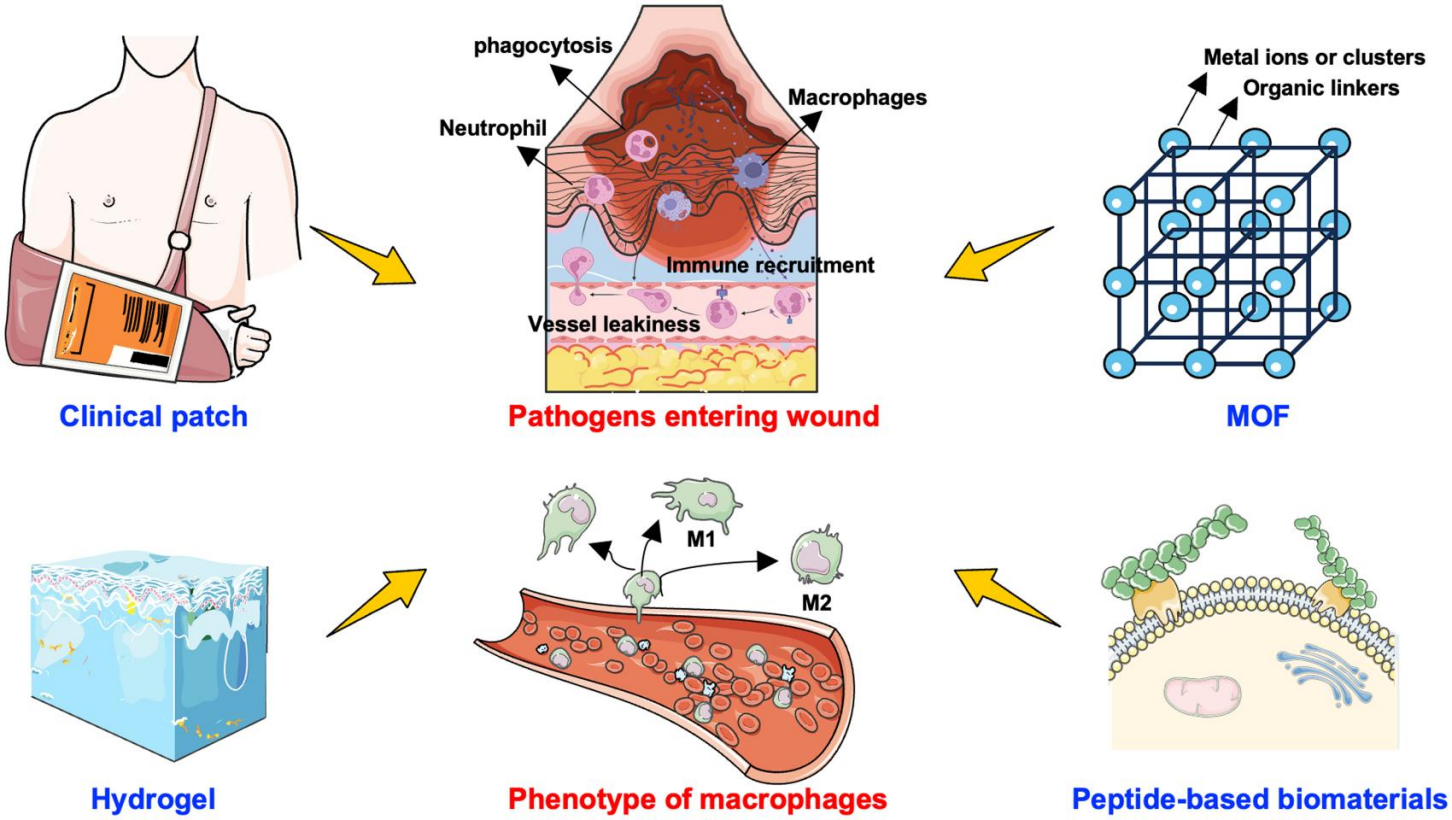
Current approaches and the future trend of designing biomaterials with the dual purpose of addressing pathogen elimination and achieving a controlled modulation of macrophage phenotypes, thereby enhancing the prospects for wound healing. Currently used materials include passive and active patches, biomimetic hydrogels, MOF and peptide-based biomaterials. Created with BioRender.com and Servier Medical Art.

The evolving landscape of wound care and continued research into biomaterials and their potential for precise immunomodulation are poised to revolutionize therapeutic approaches. Future strides in this field may lead to the development of advanced wound dressings with unparalleled capabilities, tailored to the specific needs of pathogen elimination and achieving a controlled modulation of macrophage phenotypes, thereby enhancing the prospects for wound healing (Figure 7). Additionally, the integration of cutting-edge technologies, such as metal-organic frameworks (MOF), holds great potential for personalized wound care solutions. MOFs represent a class of crystalline materials with an intriguing and versatile structure, composed of metal ions or clusters connected by organic ligands [204]. MOFs have gained considerable attention in various scientific fields due to their unique properties, such as their high surface area, tunable pore sizes and exceptional adsorption capabilities. Their ability to encapsulate therapeutic agents and release them in a controlled manner, coupled with their potential for tailored functionalization, positions MOFs as noteworthy candidates for addressing challenges in fields such as drug delivery, imaging and beyond [205]. Specifically, MOFs can serve as carriers for therapeutic agents, such as titanium carbide MXene (Ti_3_C_2_), inhibiting the growth of bacteria as well as enhancing the efficacy of wound-healing treatments [206]. Furthermore, interdisciplinary collaboration between clinicians, bioengineers and materials scientists is essential for translating these innovations from the laboratory to clinical practice.

As we move forward, a full understanding of wound biology coupled with innovative material design will shape the future of wound management, ultimately enhancing the quality of life of patients affected by acute and chronic wounds. The future of wound healing is composed of a transformative journey marked by the design of biomaterials and technological and medical innovations. Essentially, we anticipate the emergence of smart dressings with the integration of bioactive substances and real-time monitoring capabilities. Advances in regenerative medicine, such as stem cell therapies and tissue engineering, are expected to contribute to accelerated wound healing. Three-dimensional printing and nanotechnology will play pivotal roles in crafting personalized implants, scaffolds and drug delivery systems. Gene therapy holds promise for targeted genetic modifications to enhance regenerative capabilities. Artificial intelligence will revolutionize wound healing with predictive analytics and personalized treatment plans, while telemedicine and remote monitoring will facilitate efficient healthcare delivery. Patient-centric approaches, including personalized medicine and enhanced patient education, will further optimize outcomes. Collaboration, ethical considerations and regulatory approvals will be key drivers in bringing these innovations to fruition.

In conclusion, the integration of advanced materials engineering with a comprehensive understanding of wound-healing processes represents a significant leap forward in the field of wound care. As we continue to unravel the complexities of skin biology and tissue regeneration, the future of wound management holds great promise for enhancing patient outcomes and reducing the associated societal and healthcare costs.
